# Psoas Swelling in a Patient with May–Thurner Syndrome

**DOI:** 10.1055/s-0038-1675579

**Published:** 2018-10-31

**Authors:** Phillippa J. Gray, Ashu Gupta

**Affiliations:** 1 Department of General Medicine, Rockingham General Hospital, Cooloongup, Western Australia, Australia; 2 Department of Radiology, Rockingham General Hospital, Cooloongup, Western Australia, Australia

**Fig. 1:**
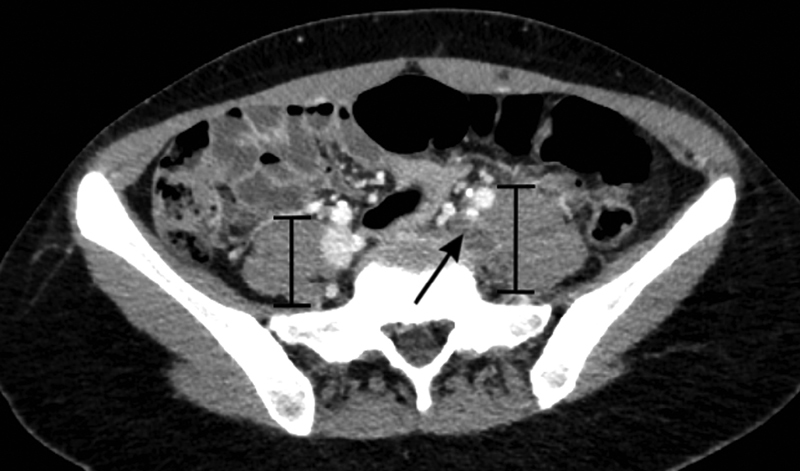
Postcontrast axial view of CT of abdomen/pelvis demonstrating a clot within the left common iliac vein ( *arrow* ) and the comparative increased size of the underlying psoas muscle ( *brackets* ).

**Fig. 2:**
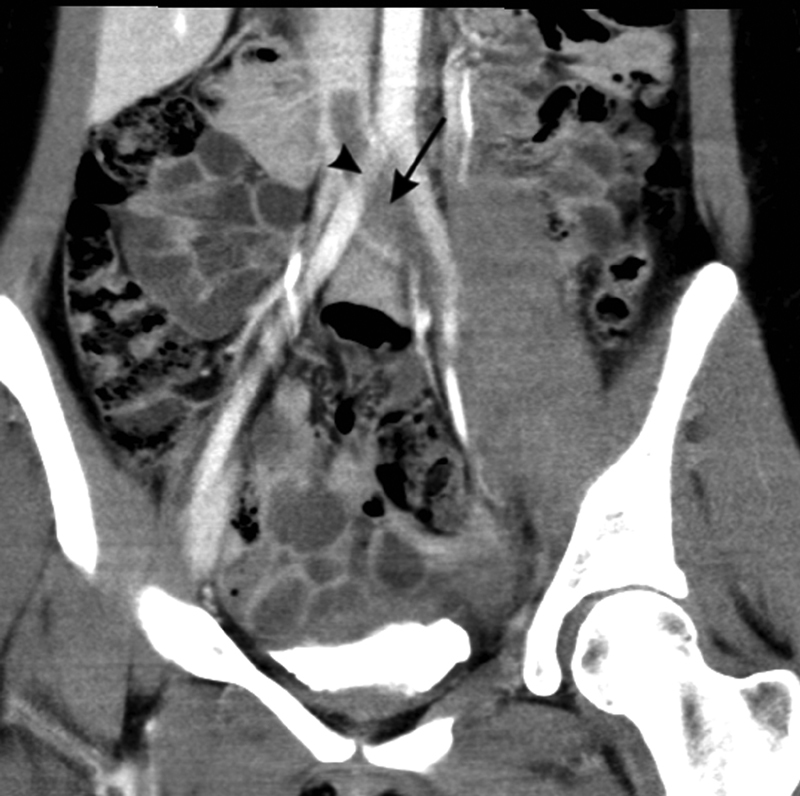
Postcontrast sagittal view of CT of abdomen/pelvis demonstrating thrombus within left common iliac vein ( *arrow* ) extending into inferior vena cava. This view also demonstrates the compression of the left common iliac vein by the overlying right common iliac artery ( *arrow head* ).

A 23-year-old female presented to the emergency department with left lower limb swelling and erythema. Ultrasound showed nonocclusive thrombus from left external iliac vein through to popliteal vein. Computed tomography of the abdomen/pelvis with contrast was performed, which shows complete occlusion and expansion of the left femoral and profunda femoris vein superiorly, the common femoral vein, and the external iliac vein in keeping with the presence of a thrombus. There was partially occlusive thrombus within the left internal iliac vein becoming occlusive toward the bifurcation. There was occlusion of the left common iliac vein, becoming partially occlusive superiorly toward the inferior vena cava (IVC). Nonocclusive thrombus was seen to extend into the IVC for approximately 3 cm. The left psoas muscle was bulky compared with the right, even above the level of the thrombus. There was mild compression of the left common iliac vein by the overlying right common iliac artery ( Figs. 1 and 2 ).

In accordance with current recommendations, she was initially discharged on rivaroxaban. 1 She re-presented to the emergency department 4 days later with sudden-onset pleuritic chest pain. As the patient was recently diagnosed with deep vein thrombosis (DVT), computed tomography pulmonary angiography was performed which revealed bilateral lower lobe pulmonary emboli. At this time, the initial thrombophilia screen came back with homozygous factor V Leiden mutation. It should be noted that thrombophilia screening should not have been indicated at this stage of the patient's care 2 ; however, the acute setting does not impact the integrity of a genetic test but may skew other results within the screening. IVC filter was placed because of high clot burden and to prevent further embolization. Percutaneous thrombolysis of femoral vein using fluoroscopic guidance and insertion of a femoral vein stent were also undertaken. She was discharged on life-long anticoagulation.

May–Thurner syndrome (MTS) refers to compression of the left common iliac vein against the fifth lumbar vertebra by the overlying right common iliac artery. This causes both mechanical compression and induction of collagen and elastin deposition resulting in intraluminal webs and “spurs” which increases the risk of clot formation. 3 The left common iliac vein is predisposed to compression owing to its more transverse course within the pelvis. MTS in particular is thought to be responsible for 18 to 49% of left lower limb DVTs. 4

On multidetector computed tomography, associated findings include lower extremity swelling, venous congestion, and perivascular inflammation, 5 which may account for the psoas findings in this case. Interestingly, pulmonary embolism is a less common complication in cases of DVT associated with MTS, due to the narrowing of the left common iliac vein trapping large emboli. 4

Follow-up ultrasound 10 months later showed patent stent and no residual thrombus. The presence of hypercoagulable states in the setting of endovenous stents is associated with higher rates of secondary loss of stent patency and stent thrombosis. This is observed in few anecdotal case reports 6 suggesting that this is an area that would benefit from further research.
